# Artificial Intelligence Technologies in Neurosurgery: a Systematic Literature Review Using Topic Modeling. Part II: Research Objectives and Perspectives

**DOI:** 10.17691/stm2020.12.6.12

**Published:** 2020-12-28

**Authors:** G.V. Danilov, M.A. Shifrin, K.V. Kotik, T.A. Ishankulov, Yu.N. Orlov, A.S. Kulikov, A.A. Potapov

**Affiliations:** Scientific Board Secretary; N.N. Burdenko National Medical Research Center for Neurosurgery, Ministry of Health of the Russian Federation, 16, 4^th^ Tverskaya-Yamskaya St., Moscow, 125047, Russia; Head of the Laboratory of Biomedical Informatics and Artificial Intelligence; N.N. Burdenko National Medical Research Center for Neurosurgery, Ministry of Health of the Russian Federation, 16, 4^th^ Tverskaya-Yamskaya St., Moscow, 125047, Russia;; Scientific Consultant, Laboratory of Biomedical Informatics and Artificial Intelligence; N.N. Burdenko National Medical Research Center for Neurosurgery, Ministry of Health of the Russian Federation, 16, 4^th^ Tverskaya-Yamskaya St., Moscow, 125047, Russia;; Physics Engineer, Laboratory of Biomedical Informatics and Artificial Intelligence; N.N. Burdenko National Medical Research Center for Neurosurgery, Ministry of Health of the Russian Federation, 16, 4^th^ Tverskaya-Yamskaya St., Moscow, 125047, Russia;; Engineer, Laboratory of Biomedical Informatics and Artificial Intelligence; N.N. Burdenko National Medical Research Center for Neurosurgery, Ministry of Health of the Russian Federation, 16, 4^th^ Tverskaya-Yamskaya St., Moscow, 125047, Russia;; Head of the Department of Computational Physics and Kinetic Equations; Keldysh Institute of Applied Mathematics, Russian Academy of Sciences, 4 Miusskaya Sq., Moscow, 125047, Russia; Staff Anesthesiologist; N.N. Burdenko National Medical Research Center for Neurosurgery, Ministry of Health of the Russian Federation, 16, 4^th^ Tverskaya-Yamskaya St., Moscow, 125047, Russia;; Professor, Academician of the Russian Academy of Sciences, Chief Scientific Supervisor N.N. Burdenko National Medical Research Center for Neurosurgery, Ministry of Health of the Russian Federation, 16, 4^th^ Tverskaya-Yamskaya St., Moscow, 125047, Russia;

**Keywords:** neurosurgery, artificial intelligence, topic modeling in neurosurgery, natural language processing

## Abstract

The current increase in the number of publications on the use of artificial intelligence (AI) technologies in neurosurgery indicates a new trend in clinical neuroscience.

**The aim of the study** was to conduct a systematic literature review to highlight the main directions and trends in the use of AI in neurosurgery.

**Methods.:**

Using the PubMed search engine, 327 original journal articles published from 1996 to July 2019 and related to the use of AI technologies in neurosurgery, were selected. The typical issues addressed by using AI were identified for each area of neurosurgery.

**Results.:**

The typical AI applications within each of the five main areas of neurosurgery (neuro-oncology, functional, vascular, spinal neurosurgery, and traumatic brain injury) were defined.

**Conclusion.:**

The article highlights the main areas and trends in the up-to-date AI research in neurosurgery, which might be helpful in planning new scientific projects.

## Introduction

In the first part of this systematic review (see Sovremennye tehnologii v medicine 2020; 12(5): 106), using topic modeling, we determined the main topics featured in publications on artificial intelligence (AI) in five important fields of neurosurgery: neuro-oncology, functional, vascular, spinal neurosurgery, and traumatic brain injury. The second part of the review presented here discusses the main issues addressed by the authors to test and evaluate AI methods.

## Methods

The review was conducted using PRISMA (Preferred Reporting Items for Systematic Reviews and Meta-Analyzes) guidelines [1].

The analysis covered journal articles and international conference proceedings that met the following criteria:

the publication was an original research article;

the publication referred to a disease and/or a treatment directly related to neurosurgery;

the authors analyzed the use of AI technology for solving clinical problems of diagnosis, treatment, prognosis, rehabilitation, or prevention of a nervous system disorder;

neurosurgery was a potential or actual field of application for the AI technology analyzed in the study.

The literature search for this systematic review was performed with PubMed US National Library of Medicine search engine system (https://www.ncbi.nlm.nih.gov/pubmed/). The search query was phrased to find all documents, in which the terms “neurosurgery” or “neurosurgical procedures” were coupled with terms related to specific AI technologies (including big data analysis and machine learning) in all database fields. The exact and complete query in the PubMed search engine is given below:

*(“neurosurgical procedures”[MeSH Terms] OR (“neurosurgical”[All Fields] AND “procedures”[All Fields]) OR “neurosurgical procedures”[All Fields] OR “neurosurgery”[All Fields] OR “neurosurgery”[MeSH Terms]) AND (“artificial intelligence”[All Fields] OR “machine learning”[All Fields] OR “natural language processing”[All Fields] OR NLP[All Fields] OR “text mining”[All Fields] OR “fuzzy logic”[All Fields] OR “data mining”[All Fields] OR “big data”[All Fields] OR “topic model”[All Fields]).*

The search results from PubMed were reviewed to select original articles that met the inclusion criteria in this systematic review. The selected articles were classified according to the relevant fields of neurosurgery. The topics in these studies were preliminary outlined by experts and their number was counted. The selection process and the technical tools used in this systematic review have been detailed in Part I.

## Results

In accordance with the inclusion criteria, 327 papers published between 1996 and July 2019 were selected. A complete list of the 327 publications is presented in the Appendix. Below, we analyze the application of AI technologies in each field of neurosurgery in more detail; typical publications illustrating the study objectives are shown in brackets.

### Application of artificial intelligence in neuro-oncology (133 publications).

 Approximately 41% of the selected studies provide examples of using AI technologies in neuro-oncology (see Appendix). Most of data for these works were obtained from medical images (magnetic resonance imaging, computed tomography, positron emission tomography, ultrasound diagnostics, optical coherence tomography, or confocal laser microscopy) as well as from genomic sequencing and histology.

The main tasks in neurosurgical oncology attempted to be solved using AI technologies were:

segmentation and volumetry of brain structures [2, 3];

noninvasive tissue and molecular genetic differential diagnosis [4–7];

predicting complications and treatment outcomes [8, 9].

One of the unconventional AI applications in neuro-oncology was the analysis of research trends in neuro-oncology based on scientific publications. An interesting task to be solved was concerned with the brain shift during neurosurgery. The solution to this problem is of great importance for the developing technologies of intraoperative neuro-navigation.

### Application of artificial intelligence in functional neurosurgery (62 publications).

 Approximately 19% of articles on the use of AI in neurosurgery address the issues of functional neurosurgery, including epilepsy surgery (see Appendix). The data in these studies were obtained from electroencephalography and electrocorticography, magnetoencephalography, medical imaging (magnetic resonance imaging, including functional imaging and resting state, positron emission tomography, single-photon emission computed tomography), as well as deep brain stimulation, video monitoring of patient’s condition, medical records, biochemical and genetic analyses, wearable devices.

Among the typical tasks approached by researchers in these studies, there were:

diagnosis of epilepsy, ictal and interictal activity, seizure predictors [10–12];

functional tractography [13];

search for epileptogenesis biomarkers [14];

patient selection for epilepsy surgery [15];

predicting outcomes of epilepsy treatment [16];

Parkinson’s disease diagnostics [17];

study of functional compensatory mechanisms in patients with Parkinson’s disease [18];

study of movement disorders in Parkinson’s disease [19];

electrophysiological identification of postural tremor and voluntary movements with essential tremor [20];

detection of targets, planning and modulating of deep brain stimulation [21];

prediction of postoperative side effects [22];

predicting the outcomes of microvascular decompression in hemifacial spasm [23];

search for electrophysiological correlates of neuropathic pain [24];

diagnosis of neuropsychiatric disorders [25];

research of episodic and semantic memory mechanisms [26];

speech-based identification of hemisphere dominance [27];

detection of the epileptogenic focus and segmentation of brain structures [28].

This section of neurosurgery is characterized by a greater variety of research tasks as compared with neuro-oncology. Predominantly, attention was paid to epilepsy and movement disorders. The principal source of data was the electrical activity of the brain as recorded not only from the scalp, but also from the cortex and deep brain structures including specific neural ensembles. Using intracranial microelectrodes, researchers were able to directly measure the electrical activity in the brain and then analyze that in studies on memory and speech.

### Application of artificial intelligence in vascular neurosurgery (44 papers).

 Research in vascular neurosurgery accounted for 14% of the entire pool of retrieved publications on AI in neurosurgery (see Appendix). Most of the data were retrieved from medical images (computed tomography, including angiography, magnetic resonance imaging, digital subtraction angiography, and X-ray images) followed by the data from medical information systems, bedside monitor recordings, electroencephalography, stereolithography, blood tests, and social/demographic questionnaires. A non-typical source of data in this field was a study on environmental pollution.

The key tasks of the above studies were:

identification of patients with aneurysms [29];

diagnosis of unruptured aneurysms [30];

risk factors and risk assessment for aneurysm rupture (including small ones) and spontaneous intracranial hemorrhage [31];

risk assessment for cardiac disorders after aneurysmal subarachnoid hemorrhage [32];

predicting outcomes of ruptured aneurysms [33];

predicting ischemia after aneurysmal subarachnoid hemorrhage [34];

grading the severity of intracranial vascular stenosis [35];

diagnosis of atherosclerotic plaques [36];

predicting a persistent decrease in blood pressure after carotid stenting and angioplasty [37];

risk assessment for stroke after carotid artery stenting [38];

predicting perfusion deficiency [39];

segmentation of blood vessels and arteriovenous malformations [40];

predicting adverse events and outcomes of treatments for arteriovenous malformations [41];

definition, classification, and segmentation of intracranial/intracerebral hemorrhage [42];

discovering the genesis of intracranial hematomas [43];

predicting an increasing volume of hypertensive intracerebral hematoma [44];

risk assessment for intracerebral hemorrhage [42];

predicting functional outcomes of intracerebral hemorrhage [45];

predicting consciousness impairment in hemorrhagic stroke [46].

Despite the substantially smaller number of publications as compared to neuro-oncology, the field of vascular neurosurgery earned a fairly wide range of AI applications. Most of these tasks can be summarized as (i) diagnosing pathological vascular formation and (ii) predicting complications and treatment outcomes.

### Application of artificial intelligence in spinal neurosurgery (29 publications).

 The use of AI in the field of spinal neurosurgery was discussed in 8% of the publications (see Appendix). The main sources of data in this area were medical images (magnetic resonance imaging, computed tomography, X-ray scans, and ultrasound images), as well as the data obtained from limb motion sensors. Special data were derived from video recordings of the surgeon’s movements, as well as articles from the US National Library of Medicine. The main tasks addressed in this area of research included:

prediction of complications [47];

prediction of treatment outcomes [48];

hospital discharge options [49];

transformation of images into different modalities [50];

segmentation of bone tissue and the spine [51];

grouping of patients and interventions for spinal deformation surgery [52];

assessing the motor function in patients with cervical spine disease and lumbar stenosis of the spinal canal [53, 54];

assessing the foot drop in lumbar radiculopathy [55]; visual diagnosis of low back pain [56];

predicting the long-term use of opioids after surgery [57];

predicting the development of osteoporosis [58].

The unusual research in this category included two studies on predicting changes in the intracranial pressure in patients during spinal surgery and predicting the surgeon’s actions during the intervention [59, 60].

### Application of artificial intelligence in the field of traumatic brain injury (26 publications).

 In 8% of the selected studies, AI was used for issues of neuro-traumatology (see Appendix). The reported data were extracted from medical images (computed tomography, magnetic resonance imaging, and terahertz neuroimaging), electroencephalography, patient monitoring devices (including intracranial pressure sensors), clinical examinations, eye movement recordings, and genetic studies.

With a relatively small pool of AI-associated articles, traumatic brain injury is an area with multiple data sources and quite a few challenging problems related to prognosis. The main objectives of the analyzed studies were:

assessing the severity of traumatic brain injury [61];

diagnosis of impaired consciousness [62];

quantitative assessment of intracerebral changes [63];

search for biomarkers of traumatic brain injury [64];

prediction of secondary injuries, classification of adverse events and complications [65];

predicting outcomes, searching for prognostic factors [66];

evaluating autoregulation of cerebral blood flow [67];

noninvasive assessment of intracranial pressure [68].

### Other publications (various and mixed topics).

 About 10% of the studies on AI application in neurosurgery were concerned with general issues relevant to a number of neurosurgical conditions or non-specific for any disease. In these studies, medical imaging data, electrophysiological data, and other sources described above were used. For example, an analysis of infectious complications in patients in the neuro-intensive care unit.

Non-standard sources of data in this area included photographs of patients’ faces, information from smartphone (to monitor neurological status), and texts of scientific publications.

### Artificial intelligence technologies used in the research.

 In the above studies, both traditional approaches to classification and prediction (regression models, support vector machines, decision trees, and the naive Bayes classifier) and the most recent methods (artificial neural networks and natural language processing) were used. Thus, almost the entire spectrum of modern AI technologies was involved in these studies.

## Discussion

According to our literature review, the majority of data concerning the use of AI technologies in neurosurgery come from neuroimaging, genome sequencing, invasive and non-invasive biosensors, and medical information systems (including unstructured data). Along with that, much less attention has been paid to the AI-based analysis of medical texts (scientific literature and medical records related to neurosurgery).

Current technologies of text analysis allow one to explore the content of medical records, highlight relevant information, and test scientific hypotheses. AI technologies can not only extract data from medical texts but also create diagnostic and predictive models based on this data [69]. An archive of electronic medical records accumulated over a long time can provide the basic material for this type of analysis. These records are currently used (with certain limitations) in retrospective studies, but their informational value is yet to be uncovered [70]. One example is the prediction of nosocomial infections following neurosurgery using Natural language processing (NLP) [71, 72]. Cohen et al. [15] showed that NLP and machine learning methods could help select candidates for surgical treatment of epilepsy and reduce the time needed to make the decision on this treatment. Generalization of risk factors for neurosurgical diseases and prediction of their clinical course by analyzing medical texts are rarely reported.

Importantly, electronic medical libraries represent an invaluable source of “big” data for research tasks. Today, generalization and critical assessment of research results using the criteria of evidence-based medicine are carried out by experts who write systematic reviews and meta-analyses [73]. As a rule, such generalization studies are labor- and time-consuming challenges; they may provide the most substantiated answers but to a very narrow range of questions. At the same time, the scientific literature of less “high quality” (from the standpoint of evidence-based medicine) contains a large amount of information that is not critically assessed by traditional evidence-based medicine methods. In surgery, including neurosurgery, the number of studies with a high level of evidence is significantly smaller than that in internal medicine [73, 74]. Therefore, analytical tools able to avoid the limitations of systematic reviews and make the most of information from thousands of published reports are of particular importance in neurosurgery. In recent years, works have appeared in which the literature on neurosurgery is analyzed and summarized using AI [75–77]. Such technologies (topic modeling) are used in this review.

Today, a common limitation of AI-assisted research in neurosurgery is a relatively small sample size (tens, hundreds, rarely thousands of observations) compared to the “big data” that has been processed by machine learning in other branches of science and industry. This data insufficiency calls into question the scalability of AI-powered solutions, even though it performs well in isolated studies. That is why research projects using AI technologies are beneficial and productive for medical research organizers who own the resource of big data.

In our opinion, when planning research using AI technologies, it is important to adhere to the principles of evidence-based medicine, which ensure the scientific validity of the obtained results and, therefore, increase their benefit to patients. Research planning and the choice of methodology are of fundamental importance and should be adequate to achieve the study objective while minimizing potential biases. In addition, technologies for analyzing large amounts of data can, to a certain extent, compensate for the methodological limitations of research in neurosurgery (for example, the inability of using randomization and double masking).

### Purpose and perspectives of artificial intelligence in neurosurgery.

With a wide range of tasks demonstrated in this review, the application of the AI technologies can be reduced to two major areas — research and clinical practice.

In research, the AI potential involves its ability to extract a lot of useful information and knowledge by making this extraction most efficient and by reducing the time of investigation. This approach overcomes the limitations of traditional mathematical statistics, which test hypotheses using much fewer data.

In clinical practice, AI can be instrumental in solving tasks of medical activities automation, i.e. accelerating, simplifying, and increasing the reliability of diagnosing diseases, making clinical decisions, and predicting possible complications and outcomes of medical interventions. Thus, AI is expected to bring an economic effect by reducing human labor costs and the time for decision-making in medicine.

A good understanding of the AI technologies perspectives justifies the efforts to develop and test these methods in research and clinical medicine.

### Interpretation of the term “artificial intelligence” in the context of medical issues.

 The term “artificial intelligence” can be intuitively (but at present, erroneously) understood as “a machine capable of thinking”, and in the context of medicine — literally “a robotic doctor”. Neuroscience has not yet reached the understanding of human intelligence, and AI technologies have not come close to creating an “artificial brain”. At present, the methods united by the concept of “artificial intelligence”, solve rather traditional and pragmatic tasks, but using entirely novel technologies. Calculations based on AI methods, like the results of traditional statistical analysis, are interpreted by a medical doctor. Despite the similarity between the principles of mathematical algorithms and human intellectual activity, the AI methods cannot yet imitate consciousness, conscience, reflection, and other abilities of the human mind, which allow the doctor to make informed and responsible decisions. Like robotic navigation systems, microscopes, and linear accelerators, AI technologies are new tools in the doctor’s arsenal. Today, they have the potential to become routine, but, for now, they cannot replace a clinician.

### Study limitations.

 This work includes a large number of publications reflecting major trends in using AI in neurosurgery without addressing the issues of efficacy, safety, or economic feasibility of specific technologies under specific conditions. Thus, the authors limit their scope to stating the facts of using AI methods in various tasks without assessing the quality of these studies.

## Conclusion

To date, research using artificial intelligence technologies has been carried out mainly in five major areas of neurosurgery: neuro-oncology, functional, vascular, and spinal neurosurgery, and the area of traumatic brain injury. The main sources of data in these studies were neuroimaging, genome sequencing, biosensors, and medical records. The use of artificial intelligence is becoming a global scientific trend in neurosurgery, therefore, an assessment of their efficacy, safety, and applicability in the clinic is highly important.
